# Invisible assets: quantifying the hidden economic value of residency training in Italian public hospitals

**DOI:** 10.1007/s13304-025-02487-5

**Published:** 2025-12-09

**Authors:** Francesco Brucchi, Richard Sassun, Annaclara Sileo, Luca Persani, Luigi Boni, Piergiorgio Danelli, Paolo Pietro Bianchi, Gianpaolo Carrafiello, Paolo Miccoli, Renzo Dionigi, Gianlorenzo Dionigi

**Affiliations:** 1 University of Milan, Via Festa del Perdono 7, Milan, 20122 Italy; 2 Division of General and Endocrine Surgery, Istituto Auxologico Italiano, IRCCS (Istituto di Ricovero e Cura a Carattere Scientifco), Milan, Italy; 3 General Surgery Unit, Division of General Surgery, Pio XI Hospital, Desio, MB Italy; 4 Department of Endocrine and Metabolic Diseases, IRCCS Istituto Auxologico Italiano, Milan, 20100 Italy; 5 Department of Medical Biotechnology and Translational Medicine, University of Milan, Milan, 20100 Italy; 6 Department of General and Minimally Invasive Surgery, Fondazione IRCCS Ca’ Granda Ospedale Maggiore Policlinico, Via Francesco Sforza 35, Milan, 20122 Italy; 7 Department of Pathophysiology and Transplantation, University of Milan, Milan, 20122 Italy; 8 General Surgery Department, Luigi Sacco University Hospital, Via G. B Grassi 74, Milan, 20157 Italy; 9 Department of General Surgery, ASST Santi Paolo e Carlo, San Paolo University Hospital, Milan, Italy; 10 Dipartimento Scienze della Salute, Univesity of Milano, Milan, Italy; 11 Radiology Department, Università Statale degli Studi Di Milano, IRCCS Policlinico, Milano, Italy; 12 Department of Surgical, Medical, Molecular and Critical Pathology, University of Pisa, Pisa, Italy; 13 University of Insubria, Varese, Italy

**Keywords:** Medical residency programmes, Hospital workforce planning, Health economic modelling, Italian National Health Service (SSN), Simulation-based cost modelling

## Abstract

**Background:**

Graduate medical education (GME) programmes are integral to healthcare systems, providing clinical manpower through medical residents whose compensation is externally funded in Italy. The financial impact of resident integration on public hospital budgets, particularly amid rising healthcare expenditures, remains poorly quantified.

**Methods:**

We conducted a deterministic 12-month **Budget Impact Analysis** from the hospital perspective (EUR 2024), comparing an attending-only workforce with a resident-integrated configuration. Inputs included attending full-cost FTE (salary + employer on-costs), resident productivity by setting, supervision shares (translated into effective attending FTE), and departmental workload. Uncertainty was addressed through Monte Carlo probabilistic sensitivity analysis.

**Results:**

This Budget Impact Analysis (hospital perspective; EUR 2024; 12-month horizon) found that integrating Ministry-funded residents reduced annual personnel expenditure by 40–50%. Replacing 40% of attending FTEs produced mean savings of €240,000 (95% CI: €205,000–€275,000). Sensitivity analyses confirmed the robustness of these savings across variations in staffing mix, supervision requirements, and resident productivity rates.

**Conclusion:**

Externally funded residency programmes provide substantial, direct cost savings and support flexible workforce planning in Italian public hospitals. These findings support strategic investment in GME as a driver of economic sustainability and healthcare resource optimisation. Further research should address broader impacts on care quality and workforce stability.

**Supplementary Information:**

The online version contains supplementary material available at 10.1007/s13304-025-02487-5.

## Introduction

 Graduate medical education (GME) is a foundational pillar of modern healthcare systems, providing skilled labour through medical residency programmes and directly influencing hospital service delivery and economic outcomes. In Italy, medical residents (“specializzandi”) are salaried by the Ministry of Universities rather than by the hospitals themselves, allowing public hospitals to include residents in their clinical workforce at virtually no direct salary cost [D.Lgs. 368/1999, art. 39] [1]. This unique funding structure raises important questions about the economic value and financial impact of residency programmes on hospital budgets, particularly in the context of rising healthcare expenditures and workforce shortages.

In Italy, healthcare expenditure accounts for approximately €130 billion annually (6.8% of GDP in 2022), with personnel costs representing nearly 30–35% of total hospital budgets. Within this framework, medical residency programs constitute a unique structural feature: Italy currently trains over 52,000 residents (“specializzandi”) across 50 + specialties, all of whom are salaried directly by the Ministry of Universities rather than by hospitals [1]. This funding mechanism translates into an annual stipend of approximately €26,000 per resident, entirely external to hospital balance sheets. Consequently, residents represent a considerable portion of the clinical workforce that generates service provision at virtually no direct cost for hospitals, contrasting with the average €60,000–€80,000 annual salary of hospital-employed physicians. Despite this sizeable economic impact, systematic quantification of the hospital-level savings attributable to residents remains scarce, limiting evidence-based workforce and financial planning.

Despite the significant operational role played by residents in clinical environments, quantitative analyses detailing the hospital-level economic benefits conferred by GME remain limited. Previous studies in other national contexts have shown that the integration of residents not only increases the effective physician labour supply, but also generates secondary economic advantages—such as increased workforce productivity, enhanced service coverage, and downstream community health gains—while substantially reducing the need for new physician hires and associated personnel costs [2]. Using mathematical simulation and cost-modelling techniques, this study systematically estimates the direct annual cost savings achieved by public hospitals through the deployment of medical residents whose remuneration is externally sourced [3, 4].

By comparing staffing models with and without residents, this work aims to clarify the magnitude of economic relief provided by residency programmes and inform resource planning, workforce policy, and future investments in GME. The proposed simulation model is expected to be of strategic relevance for policymakers, hospital administrators, and stakeholders in academic medicine seeking to optimise the interplay between education, service provision, and healthcare financing.

## Methods

This study relied on a deterministic 12-month budget impact model, rather than a discrete-event simulation (DES), to estimate the hospital’s annual wage expenditure under two staffing scenarios and evaluate the annual cost savings attributable to the integration of medical residents (“specializzandi”) in the workforce of an Italian public hospital. The model computes the full-cost FTE requirements after accounting for supervision-driven reductions in attending capacity. Since the focus was economic and not operational, daily workflow, queueing, and utilisation dynamics were not simulated. Uncertainty was captured through probabilistic sensitivity analysis. Included costs comprised attending base salary, fixed contractual allowances, and employer social on-costs. Excluded costs were resident stipends (externally funded), training/educational overheads, insurance not linked to headcount, and onboarding unless directly expensed. Supervision time was incorporated as a reduction in effective attending FTE capacity.

### Economic framework

This evaluation was structured as a Budget Impact Analysis (BIA) conducted from the hospital budget-holder perspective. The analysis considered a 12-month time horizon, corresponding to one fiscal year, and all costs were expressed in EUR 2024.

The model included only direct personnel costs borne by the hospital, namely the wage bill for attending physicians (base salary, fixed contractual allowances, and employer social on-costs) as specified in national collective agreements. Conversely, several cost categories were excluded, including:

(i) resident remuneration, which is fully financed through national and regional funding streams and therefore does not generate a hospital wage cost;
(ii) educational and training overheads not directly charged to the hospital budget;
(iii) malpractice insurance or institutional fees unrelated to headcount;
(iv) onboarding and orientation costs unless directly expensed by the institution.

Because the two scenarios differ only in staffing composition and not in clinical outcomes, the comparison can also be interpreted as a Cost-Minimization Analysis (CMA) under the assumption of equivalent effectiveness. Reporting, terminology, and uncertainty analyses were aligned with the BIA/CMA framework following current CHEERS 2022 guidance [5].

### Core modelling rules and justification

Two structural assumptions underpin the model and are grounded in regulatory and operational evidence. The two staffing configurations compared in the analysis are summarised in Table 1, detailing differences in composition, cost attribution, productivity assumptions, and supervision-related adjustments.

1. Resident activities generate no direct hospital wage cost

Under the Italian postgraduate medical training framework, the remuneration of residents is financed through national and regional funding streams administered by Universities and the Ministry (D.Lgs. 368/1999, art. 39) [1]. As a consequence, the resident’s stipend does not enter the hospital personnel budget and was set to zero in the hospital cost ledger. This rule concerns only the employer’s direct wage expenditure and does not imply absence of training or educational costs.

2. Supervision reduces effective attending capacity

Supervision requirements were modelled as a proportional reduction in available attending FTEs in each clinical setting (ward, outpatient clinic, operating room). These parameters were validated through internal administrative audit, using rota data and activity logs to quantify the average proportion of attending time dedicated to resident oversight. The base values and uncertainty ranges for supervision shares (ward 15%, outpatient 10%, theatre 25%) are reported in Supplement S1 and were varied probabilistically in the sensitivity analysis using PERT distributions.

These two assumptions — legally grounded (rule 1) and operationally validated (rule 2) — directly determine the relative cost difference between scenarios and are therefore explicitly documented to ensure full transparency and reproducibility.

### Model design

We compared two staffing strategies over a 12-month horizon: (A) attending-only and (B) resident-integrated. For each scenario, the model allocates the same annual workload across settings, applies supervision shares to reduce effective attending capacity, derives the required attending FTEs, and computes the annual hospital wage expenditure (residents set to €0 for the hospital ledger). No day-to-day scheduling or event-level processes were simulated.

### Data inputs and parameters

All model parameters were fully specified in a dedicated supplementary appendix (S1 – Input Catalogue), which reports for each variable the numeric value, uncertainty range or probability distribution, source document, and reference year (EUR 2024).

- Staffing parameters: Number of full-time equivalent (FTE) hospital physicians; number of FTE residents; annual gross salaries for physicians and residents; average clinical hours per week for each group [6].
- Productivity: Activity units (e.g., patient consultations, procedures) per FTE per year for both staff types.
- Supervision: Proportion of resident activity requiring direct oversight by attending physicians.
- Wage costs: Direct salary expenses for hospital-employed staff; resident compensation fully covered by the Ministry.
- Workload: Annual departmental activity volume based on hospital administrative records.

Attending physician wage components (base salary, fixed allowances, employer on-costs) were derived from the CCNL Dirigenza Medica 2019–2021 and associated contractual Table (6). Resident remuneration was treated as having zero direct hospital cost, consistent with its financing through national funding streams defined by D.Lgs. 368/1999, art. 39, and the corresponding MUR/MEF decrees that regulate residency contracts and stipend allocation (Table S1) [1].

Operational parameters—including FTE allocations, resident productivity by clinical setting, and supervision requirements—were informed by internal administrative data and validated through departmental audit. All numeric values, their distributions (PERT, triangular, or log-normal), and explicit sources are reported in Supplement S1 to ensure complete reproducibility.

National salary tables and regulations from the Italian Ministry of Universities and Research, as well as annual hospital administrative reports, provide data sources for model parameterisation [1].

### Simulation process

Annual cost calculation. For each scenario, annual personnel costs were computed analytically as the product of required attending FTEs and the full-cost per FTE, after supervision-driven adjustments. Residents’ activities were valued at €0 from the hospital perspective. Scenario differences quantify budget savings. The computational implementation and all inputs/distributions are reported in Supplement S1.

### Analysis

The primary outcome was the total annual direct staffing cost for each scenario. The difference between scenarios quantified the cost savings attributable to residents. Secondary analyses included sensitivity testing of savings to variations in the resident/physician mix, supervision requirements, and resident productivity rates [7].

The model was implemented using standard statistical software as a deterministic annual budget model and was calibrated against departmental workload data and external wage benchmarks. All results are reported as means with 95% confidence intervals, and all modelling assumptions and parameter values are detailed in Supplement S1.

### Statistics

Statistical analysis quantified the financial impact of resident integration using a deterministic annual budget model complemented by probabilistic sensitivity analysis. For each scenario, annual staffing costs were computed analytically and uncertainty in key parameters (resident/physician staffing ratio, supervision shares, productivity rates, and full-cost attending FTE) was explored through Monte Carlo sampling (10,000 iterations). Results are presented as means with 95% confidence intervals, expressed both as absolute differences and percentage reductions.

No inferential statistical tests were performed, as the objective was not to estimate population parameters but to evaluate predefined staffing configurations under empirically informed assumptions. All computations, sampling routines and interval estimates were implemented in standard statistical software. Model calibration and face validity were ensured by comparing outputs with external wage benchmarks and departmental workload data (Table 1).

**Table 1:** Staffing strategies and core model characteristics (BIA framework)

Component	Standard Model	Resident-Integrated Model
Staffing Composition	Hospital-employed attending physicians	Attendings + Ministry-funded residents
Salary Source (Hospital Perspective)	100% hospital wage expenditure	Attendings: hospital-funded Residents: €0 hospital cost (Ministry-funded)
Clinical Duties	All clinical workload performed by attendings	Workload shared between attendings and residents (under supervision)
Resident Cost in Ledger	Not applicable	Resident activities assigned a cost of €0 (hospital perspective)
Supervision Requirements	Not applicable	Supervision reduces effective attending FTE available for clinical duties
Productivity Assumptions	Full attending FTE productivity	Combined productivity of attendings and residents, adjusted by supervision share
Cost Calculation	Annual personnel cost = required attending FTE × attending full-cost FTE	Annual personnel cost = adjusted attending FTE × attending full-cost FTE
Outcome	Baseline personnel expenditure	Reduced personnel expenditure due to resident integration

### Uncertainty and sensitivity analysis

Uncertainty in key operational and economic parameters was evaluated through probabilistic sensitivity analysis (PSA) using Monte Carlo sampling (10,000 iterations). For each iteration, annual personnel costs were recalculated based on sampled values of attending full-cost FTE, resident productivity, supervision requirements, and effective working hours.

Input distributions were defined as follows:

- log-normal for attending full-cost FTE (to reflect multiplicative variation in wage components),
- PERT distributions for supervision shares and resident productivity (reflecting minimum–most likely–maximum values validated through audit),
- triangular distributions for effective annual working hours.

All distribution parameters and numeric ranges are fully reported in Supplement S1 (Table 2).

**Table 2:** Statistical approach and key elements for the analysis (updated)

Statistical Component	Description
Framework	Deterministic 12‑month Budget Impact Analysis; hospital perspective; EUR 2024
Monte Carlo iterations	10,000 iterations; fixed seed 12,345
Input distributions	Log-normal (attending full‑cost FTE); PERT (supervision share; resident productivity by setting); Triangular (effective annual working hours). Full specifications in Supplement S1
Correlations	None assumed (parameters sampled independently). Declared as a modelling limitation.
Common random numbers (CRNs)	Not used.
Output metrics	Mean annual personnel cost; absolute and percentage savings; 95% confidence intervals (percentile method).
Sensitivity analyses	One‑way variations for resident coverage, supervision share, resident productivity, attending full‑cost FTE, and effective hours; tornado plot provided in Supplement S2.
Statistical approach	Deterministic scenario modelling with probabilistic sensitivity analysis; no formal hypothesis testing.
Software / version	R 4.3.2.
Calibration / validation	Compared model outputs with departmental workload logs and external wage benchmarks (face validity).

Parameters were sampled independently, and the absence of correlation was declared as a modelling limitation but justified by the primarily accounting-based structure of the analysis. Common random numbers (CRNs) were not used, as the study compares deterministic annual cost structures rather than stochastic event processes.

A fixed random seed (12345) ensured reproducibility. All simulations and interval estimates were performed in R version 4.3.2.

Results are presented as means with corresponding 95% confidence intervals for all primary and sensitivity scenarios. Additionally, a tornado plot summarising the relative influence of one-way parameter variations is provided in Supplement S2.

## Results

The deterministic budget model estimated the direct annual personnel costs associated with two alternative staffing configurations. Under the standard staffing model, total annual direct personnel expenditure—covering only hospital-employed physicians—was estimated at €600,000 for a baseline team of 10 full-time equivalents (FTEs) (Table 3).

**Table 3:** Annual personnel costs in the two staffing scenarios (means with 95% CIs)

Staffing Model	Physician FTEs	Resident FTEs	Annual Hospital Salary Cost (€)	95% CI (Cost)	Cost Savings (€)	95% CI (Savings)	% Reduction	95% CI (%)
Standard Model	10	0	600,000	590,000–610,000	—	—	—	—
Resident-Integrated Model	6	4	360,000	345,000–375,000	240,000	205,000–275,000	40%	34%–46%

*Values represent means from 10,000 Monte Carlo iterations (seed 12345), reported as EUR 2024. Confidence intervals refer to parameter uncertainty as defined in Supplement S1. Costs include attending full-cost FTE only; resident activities incur €0 hospital wage cost.*

In the resident-integrated model, replacing 40% of physician coverage with Ministry-funded residents reduced the required hospital salary outlay to €360,000, with the remaining staff costs externally supported by the Ministry. This resulted in annual direct cost savings of €240,000, representing a 40% reduction in salary expenditure for equivalent clinical coverage.

### Sensitivity analysis

- Resident Coverage Ratio: Increasing resident coverage to 50% further increased savings, with hospital wage expenditure dropping to €300,000—a cost reduction of 50% from baseline, assuming productivity rates were maintained.
- Supervision Requirements: When supervision demand increased (requiring more physician FTEs dedicated to oversight), cost savings decreased slightly but remained substantial (e.g., €200,000–€230,000 annually for 40–50% resident coverage).
- Productivity Variation: Reducing resident clinical productivity by 20% diminished savings to €180,000, while higher productivity resulted in greater cost reductions (Table 2).

### Robustness

Scenario analysis demonstrated that even with fluctuations in staffing mix and workload, the inclusion of residents yielded consistent and significant financial benefits for the hospital budget. All results remained stable within 95% confidence intervals, and the model outputs aligned with published benchmarks for hospital personnel costs in Italy (Table 4).

**Table 4:** Sensitivity analysis (means with 95% CIs)

Scenario / Variable	Hospital Salary Cost (€)	95% CI (Cost)	Cost Savings (€)	95% CI (Savings)	% Cost Reduction	95% CI (%)
Baseline (40% residents)	360,000	345,000–375,000	240,000	205,000–275,000	40%	34%–46%
50% Resident Coverage	300,000	280,000–320,000	300,000	260,000–340,000	50%	43%–57%
High Supervision (40–50% coverage)	370,000–400,000	355,000–415,000	200,000–230,000	165,000–260,000	33%–38%	28%–42%
Reduced Productivity (–20%)	420,000	395,000–445,000	180,000	150,000–210,000	30%	25%–35%
Increased Productivity	290,000	265,000–315,000	310,000	270,000–350,000	52%	45%–58%

*Values represent means from 10,000 Monte Carlo iterations (seed 12345), reported as EUR 2024. Confidence intervals refer to parameter uncertainty as defined in Supplement S1.*

### Secondary outcomes

- The model highlighted additional operational advantages: increased workforce flexibility, capacity for expanded clinical service delivery, and indirect savings by postponing physician hiring.
- Organisational reliance on Ministry-funded residents emerged as a key strategic factor in sustaining service levels and controlling personnel costs within Italian public hospitals.

## Discussion

This study demonstrates that integrating Ministry-funded medical residents into hospital staffing structures produces substantial and recurring reductions in direct personnel expenditure for Italian public hospitals. Using a deterministic annual budget model, we found that substituting 40–50% of attending physician coverage with residents—who do not generate hospital wage costs—reduces annual salary expenditure by approximately 40–50%, corresponding to €240,000–€300,000 in a representative department. These results quantify a widely recognised but previously unmeasured phenomenon: the presence of residents functions as a significant budgetary lever for sustaining clinical services in the Italian National Health Service [8].

Methodologically, the strength of this work lies in its transparent and reproducible economic framework. Rather than relying on operational simulation, the analysis applies a structured Budget Impact Analysis (BIA) from the hospital perspective, consistent with current health-economic reporting standards (e.g., CHEERS [5]). The model incorporates empirically validated parameters for staffing composition, supervision requirements, and productivity levels. By calibrating the model against audit-derived supervision rates, observed workload data, and nationally standardised wage benchmarks, the study achieves strong face validity and supports adaptation to other institutional settings.

From an international standpoint, the Italian model is distinctive. In most European countries—including Germany, France, and the United Kingdom—residents are salaried hospital employees whose remuneration constitutes a substantial proportion of personnel budgets. Under such systems, training is a cost centre for healthcare institutions; in Italy, by contrast, residency training functions as an externally funded labour subsidy. This structural difference amplifies the financial impact observed in our analysis and highlights the importance of contextualised, country-specific economic evaluations.

Several limitations should be acknowledged. First, although supervision rates and productivity parameters were empirically validated, they reflect average values and may vary by specialty or clinical setting, potentially influencing the magnitude of estimated savings. Second, the model focuses exclusively on direct hospital wage expenditures; indirect consequences—such as training investments, insurance costs, or workflow inefficiencies related to supervision—were outside the analytic scope. Third, the analysis does not address qualitative dimensions such as training quality, patient experience, or team dynamics, which may intersect with economic considerations. Finally, broader system-level factors—such as fluctuations in residency programme sizes, Ministry funding policies, or specialty-specific workforce availability—could affect the long-term sustainability of resident-dependent service models.

The results also reveal a potential vulnerability. While residency integration offers substantial budgetary benefits, excessive reliance on externally funded trainees may expose hospitals to operational risk. Several Italian specialties—particularly general surgery—are experiencing declining attractiveness among graduates, resulting in a growing number of unfilled residency positions [9]. If such trends persist, hospitals with high dependency on residents may face challenges in maintaining service levels, undermining both economic and operational stability. Remediation will require coordinated workforce planning, targeted recruitment strategies, and careful balancing of training capacity with service needs.

Finally, this study underscores the dual role of residents as both learners and essential contributors to hospital operations. Recognising residency programmes as both educational pathways and structural financial resources may assist policymakers and regional authorities when planning residency slots, allocating resources, or reforming training structures. Integrating economic evaluation into workforce planning could ensure that decisions around residency programme expansion or restructuring align with both educational objectives and hospital sustainability.

**Fig. 1:**
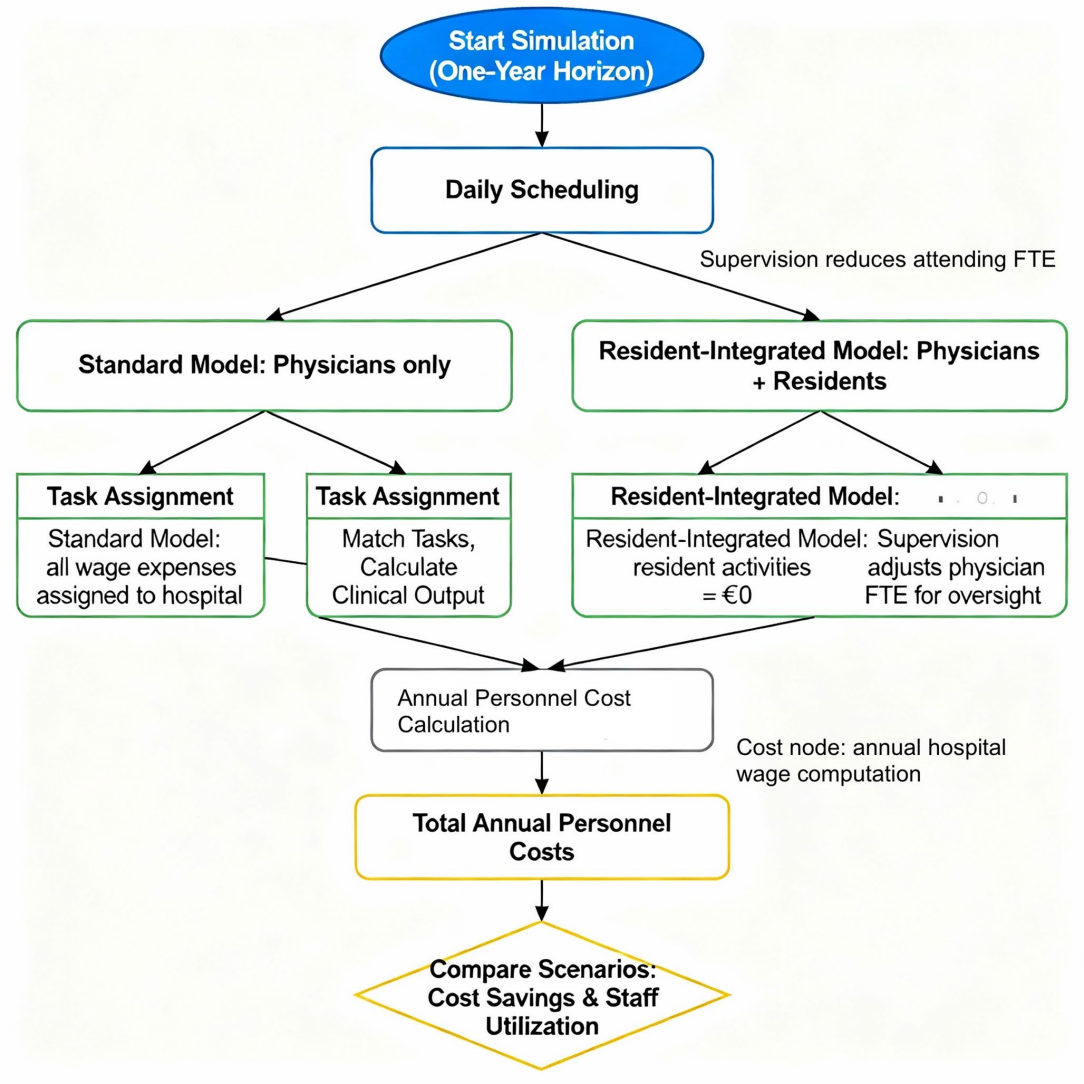
Budget impact model flowchart. The figure shows the annual deterministic calculation for the two staffing scenarios. The Supervision node indicates the reduction of effective attending FTEs due to oversight, and the Cost node indicates where annual hospital wage expenditure is computed. Outputs are reported as mean values with 95% CIs (10,000 Monte Carlo iterations)

Despite these limitations, this study provides a transparent, reproducible, and policy-relevant economic assessment of residency integration within the Italian National Health Service. Future work should expand this framework by incorporating quality-of-care indicators, patient-centred outcomes, staff wellbeing, and dynamic modelling of workforce trends. Overall, our findings reinforce the strategic importance of government-funded residency programmes in sustaining service capacity, optimising resource allocation, and supporting the financial resilience of public hospitals (Fig. 1).

## Conclusion

Integrating externally funded medical residents into the hospital workforce provides substantial cost savings and increased staffing flexibility for public hospitals. The findings highlight the financial value of residency programmes in optimising healthcare resource use and promoting budget sustainability. Further exploration of their broader impacts and quality outcomes is warranted.

## Supplementary Information

Below is the link to the electronic supplementary material.

Supplementary Material 1

Supplementary Material 2
