# Supplementary material for: Invisible assets: quantifying the hidden economic value of residency training in Italian public hospitals
Source: Updates Surg. 2025 Dec 9;78(2):961–8. doi: 10.1007/s13304-025-02487-5 (PMC13212412; doi:10.1007/s13304-025-02487-5)
Supplement: Supplementary file 1 — Supplementary Material 1 [file 13304_2025_2487_MOESM1_ESM.docx]

# Table S1a. Economic & Contractual Inputs (National Standards)

| Parameter | Base value | Distribution (parameters) | Range / Notes | Source | Year |
| --- | --- | --- | --- | --- | --- |
| Attending physician – annual gross per FTE | €60,996 | Log-normal (μ=ln(60,996); σ=0.05) | Includes base salary + fixed contractual allowances | ANAAO – CCNL 2019–2021 wage tables | 2024 |
| Employer social on-costs (INPS – CPS) | 23.8% | Fixed | Standard employer rate | INPS Public Sector Contributions | 2024 |
| INAIL premium (employer) | 1.0% | Triangular (0.5; 1.0; 1.5) | Healthcare risk category | INAIL sector benchmarks | 2024 |
| Total employer on-cost rate | 24.8% | Derived | Combination INPS + INAIL | Derived from INPS/INAIL | 2024 |
| Full-cost per FTE (gross × on-cost) | €76,123 | Log-normal (consistent with above) | Calculated as 60,996 × 1.248 | Derived calculation | 2024 |
| Resident stipend (hospital ledger) | €0 | Fixed | Externally funded; no hospital wage cost | D.Lgs. 368/1999 Art. 39 | 2024 |

# Table S1b. Operational Inputs (Audit-Validated)

| Parameter | Base value | Distribution | Range / Notes | Source | Year |
| --- | --- | --- | --- | --- | --- |
| Resident productivity – Ward | 1,200 units/yr | PERT (900;1200;1500) | Activity units per year | Internal audit – ward logs | 2024 |
| Resident productivity – Outpatient | 900 visits/yr | PERT (700;900;1100) | Annual visits | Internal audit – clinic logs | 2024 |
| Resident productivity – Theatre | 180 cases/yr | PERT (120;180;240) | Assisted OR cases | Internal audit – OR registry | 2024 |
| Supervision share – Ward | 15% | PERT (10;15;20) | Share of attending FTE consumed | Rota & supervision audit | 2024 |
| Supervision share – Outpatient | 10% | PERT (5;10;15) | Varies by teaching load | Audit | 2024 |
| Supervision share – Theatre | 25% | PERT (20;25;35) | Higher intensity in OR | Audit | 2024 |
| Effective working hours – Attending | 1,760 h/yr | Triangular (1680;1760;1840) | Contracted hours – leave | HR policy | 2024 |
| Effective working hours – Resident | 2,000 h/yr | Triangular (1800;2000;2200) | Training rota | Residency programme | 2024 |
