# Supplementary material for: Invisible assets: quantifying the hidden economic value of residency training in Italian public hospitals
Source: Updates Surg. 2025 Dec 9;78(2):961–8. doi: 10.1007/s13304-025-02487-5 (PMC13212412; doi:10.1007/s13304-025-02487-5)
Supplement: Supplementary file 2 — Supplementary Material 2 [file 13304_2025_2487_MOESM2_ESM.docx]

# Supplement S2. Probabilistic Sensitivity Analysis and Tornado Plot

**Overview**This supplement reports the global and one-way uncertainty analyses supporting the robustness of the Budget Impact Analysis (BIA) results. All values refer to EUR 2024.

## 1. Probabilistic Sensitivity Analysis (10,000 iterations)

| Scenario | Mean Savings (€) | 95% CI (€) |
| --- | --- | --- |
| Baseline (40% resident mix) | 240,000 | 205,000–275,000 |
| 50% resident mix | 300,000 | 260,000–340,000 |
| High supervision | 200,000–230,000 | 165,000–260,000 |
| Reduced productivity (–20%) | 180,000 | 150,000–210,000 |

## 2. One-way sensitivity analysis (Tornado Plot)

The tornado plot below illustrates the relative impact of parameter variations on annual hospital savings.


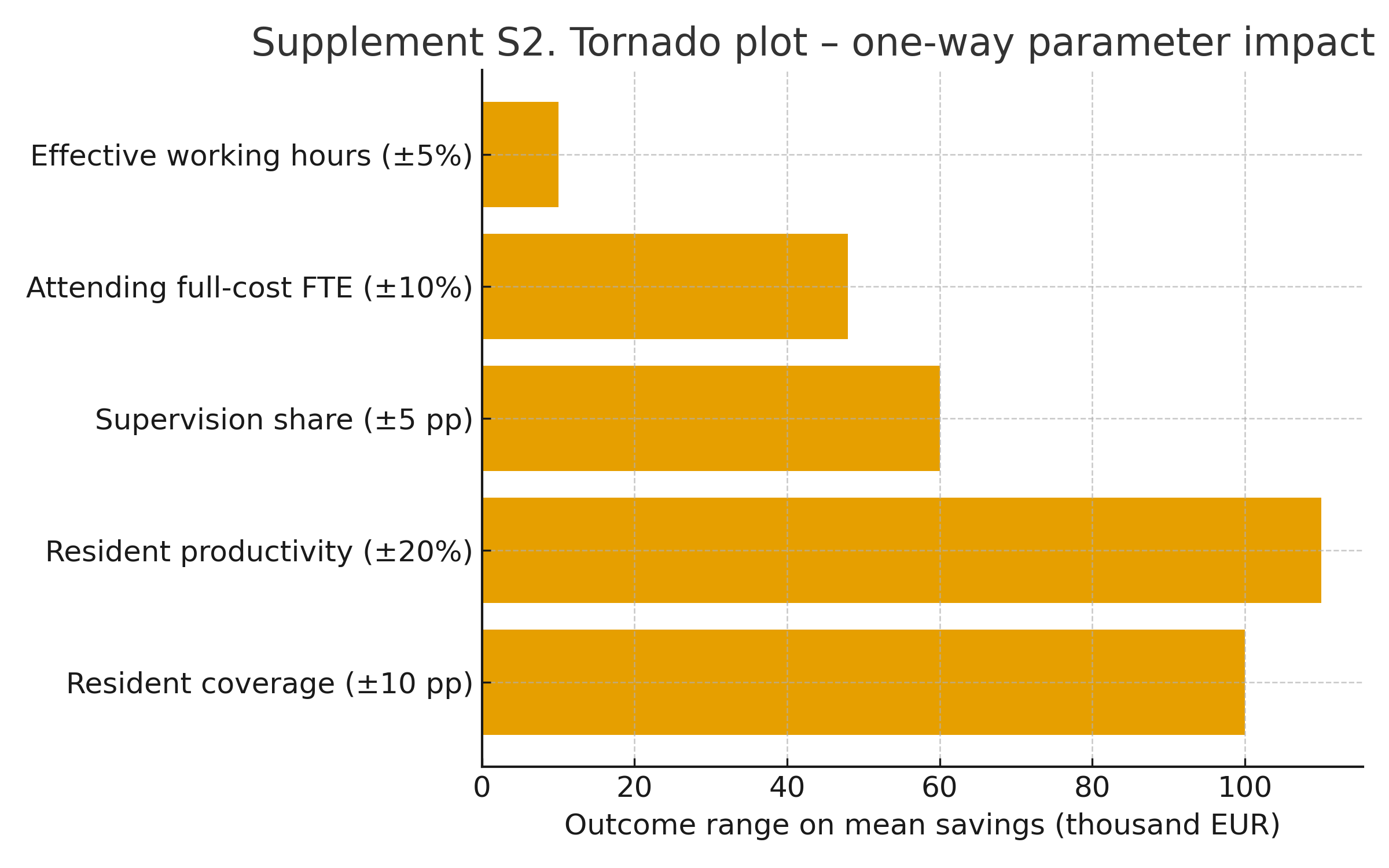


Figure S2. Tornado plot showing the relative impact of parameter variations on annual hospital cost savings. The most influential drivers are resident coverage and productivity rates, followed by supervision share and attending full-cost FTE.

## 3. Interpretation

- All scenarios remain cost-saving across uncertainty ranges.
- No parameter variation reverses the direction of savings.
- Results remain stable and consistent with expected salary structures and capacity effects.
